# Current status of inherited pancreatic cancer

**DOI:** 10.1186/s13053-022-00224-2

**Published:** 2022-06-27

**Authors:** Marek Olakowski, Łukasz Bułdak

**Affiliations:** 1 Department of Gastrointestinal Surgery, Medical University of Silesia, Medyków 14, 40-752 Katowice, Poland; 2 Department of Internal Medicine and Clinical Pharmacology, Medical University of Silesia, Medyków 18, 40-752 Katowice, Poland

**Keywords:** Pancreatic cancer, Hereditary syndromes, Familial pancreatic cancer, Mutation, Screening

## Abstract

**Background:**

It is estimated that about 10% of pancreatic cancer cases have a genetic background. People with a familial predisposition to pancreatic cancer can be divided into 2 groups. The first is termed hereditary pancreatic cancer, which occurs in individuals with a known hereditary cancer syndrome caused by germline single gene mutations (e.g., *BRCA1/2*, *CDKN2A*). The second is considered as familial pancreatic cancer, which is associated with several genetic factors responsible for the more common development of pancreatic cancer in certain families, but the precise single gene mutation has not been found.

**Aim:**

This review summarizes the current state of knowledge regarding the risk of pancreatic cancer development in hereditary pancreatic cancer and familial pancreatic cancer patients. Furthermore, it gathers the latest recommendations from the three major organizations dealing with the prevention of pancreatic cancer in high-risk groups and explores recent guidelines of scientific societies on screening for pancreatic cancers in individuals at risk for hereditary or familial pancreatic cancer.

**Conclusions:**

In order to improve patients’ outcomes, authors of current guidelines recommend early and intensive screening in patients with pancreatic cancer resulting from genetic background. The screening should be performed in excellence centers. The scope, extent and cost-effectiveness of such interventions requires further studies.

## Introduction

Recent epidemiological data show that pancreatic cancer (PC) incidence is still rising, especially in high- and very high-income countries, reaching 495,773 new cases worldwide in 2020 [1]. This translates into the incidence rate of PC around 5.7 cases per 100,000 people in males and 4.1 cases per 100,000 people in females. What-is-more, the incidence of new cases is nearly in parallel with the death toll in 2020 attaining 466,003 patients. Risk factors for developing PC can be divided into two categories: nonhereditary, which are dependent on lifestyle and environmental impact on the human body; and hereditary, which are connected with the genetic background in an individual [2]. Nowadays, it is estimated that approximately 10% of PC cases are connected with genetic factors [3]. PC may occur in individuals diagnosed with hereditary cancer syndromes caused by germline gene mutations (e.g., *BRCA1/2*, *CDNK2A*/p16). Those cases are responsible for 3% of all PC and have been denoted as hereditary pancreatic cancer (HPC) group. Another 7% of patients with PC have a significant family history (⩾2 first-degree relatives with PC). In most of these families, the germline mutation responsible for the development of PC is unknown or does not cause hereditary cancer syndrome. Individuals from such families have been categorized into the familial pancreatic cancer (FPC) group [4]. Interestingly, recent data published by the Pancreatic Cancer Case-Control Consortium (PanC4) showed that the heritability of PC might be more than twice as high as previously calculated, reaching 21.2% [5]. The importance of such findings is reflected by guidelines prepared by the European Registry of Hereditary Pancreatitis and Familial Pancreatic Cancer (EUROPAC) (Table 1), which provide clinical features of PC that raise suspicion of hereditary or familial origin. Patients suspected to have a potential germline mutation should be referred for genetic counseling [6]. This is an essential step in the introduction of specific genetic testing and prophylactic measures in these cases.

**Table 1:** European registry of hereditary pancreatitis and familial pancreatic cancer criteria [6]

EUROPAC criteria	
Criterion 1	≥2 first-degree relatives with PC
Criterion 2	≥ 3 relatives with pancreatic cancer
Criterion 3	Possible associated cancer syndrome (defined as sub-criteria below) in addition to the case of PC being studied
Criterion 3.a: HBOC	Personal/family history (≥1 first/second-degree relatives) of breast/ovarian cancer
Criterion 3.b: FAMMM	Personal/family history of melanoma in ≥1 first/second degree relative AND a high total body naevi count (often > 50)
Criterion 3.c: LS	Personal/family history (≥1 first/second-degree relatives) of a LS-associated cancer (such as colorectal, endometrial, small bowel, renal)
Criterion 3.d: PJS	Oral/mucous membrane pigmentation +/− a personal/family history (≥1 first/second-degree relatives) of gastrointestinal cancers in first/second

Abbreviations:

*EUROPAC* European Registry of Hereditary Pancreatitis and Familial Pancreatic Cancer

*FAMMM* Familial atypical multiple mole melanoma

*HBOC* Hereditary breast-ovarian cancer syndrome

*LS* Lynch syndrome

*PC* Pancreatic cancer

*PJS* Peutz-Jeghers syndrome

## Genetic syndromes associated with HPC

### Hereditary breast and ovarian cancer (HBOC)

HBOC is caused by germline loss-of-function mutations in one of the two tumor suppressor genes *BRCA1* and *BRCA2*. The product of each of these genes interacts with recombination/DNA repair proteins in pathways that are involved in maintaining intact chromosome structure and thus play an important role in the supervision of cell division. Loss of their function results in genomic instability and promotes cancer development [7]. *BRCA1* and *BRCA2* mutation carriers have a very high risk of breast cancer and ovarian cancer by age 70, at 47–66% and 40–57%, respectively, and of other malignancies, including PC [8]. PC is the third most common cancer associated with *BRCA1* and *BRCA2* gene mutations [9]. In a prospective study of 5149 female *BRCA1* and *BRCA2* mutation carriers, a statistically significant 2.4-fold increase in the incidence of PC was observed. It was calculated that the annual risk of developing PC, for women over 50 years of age with a mutation in either of these genes, is 0.04% [10]. In a retrospective cohort of 5799 families (men/women) from the HBOC group, an increased risk of PC was observed in breast and ovarian cancer patients both carrying *BRCA1* and *BRCA2* gene mutations. But the elevated risk for PC was also noted in patients with breast cancer with a negative test for *BRCA1* and *BRCA2* mutations. In families with *BRCA2* mutations in which one member had an early onset of PC (< 50 years of age), the risk of this cancer in subsequent generations was estimated to be 9.9 times higher than that in the general population [11]. It was also observed that *BRCA*-mutated PC occurs later than *BRCA*-mutated breast cancer (age: breast vs. pancreas, 45.0 vs. 53.5 years, *p* = 0.050). Therefore, active genetic testing to identify *BRCA1/2* mutation carriers at the onset of breast cancer and continuous long-term monitoring of these patients can provide opportunities to detect *BRCA*-mutated PC at a resectable stage [12].

Phenotypic variability that depended on the type of *BRCA2* mutation and family ethnicity has been documented in a study evaluating the incidence of 7 cancer types in 440 families from various countries [13]. In *BRCA2* mutation families of Polish ancestry (*n* = 26), a lower incidence of PC was observed than in families of other ethnicities (*n* = 356). The association of *BRCA2* germline mutations with HPC is well established, but the role of *BRCA1* mutations is less clear. Some authors [14] have shown that the germline *BRCA1* mutations probably predisposes patients to the development of PC, while others have argued for the absence of such a relationship [15, 16]. In a study of 2167 patients with metastatic PC from 3 countries, the presence of germline *BRCA1* and/or *BRCA2* mutations was found in 9.5% of US, 7.6% of French and 7.4% of Israeli individuals [17]. Patients with *BRCA1* and/or *BRCA2* mutations are slightly younger (57.9 vs 61.1 years) and more likely to have early-onset PC than patients without known *BRCA1* and/or *BRCA2* mutations. The reported prevalence of newly identified *BRCA1* and/or *BRCA2* mutations seem to be highest in African-American patients (10.7%) compared to Caucasian (6.1%), Asian (5.0%) and other (1.6%) patients. The impact of germline *BRCA* mutation on the survival of PC patients remains a subject of research. Positive results have been reported with the use of targeted therapies, particularly poly-(ADP-ribose) polymerase inhibitors, in *BRCA*-mutated ovarian and breast cancers, and their use is currently being investigated in germline-mutated PC [18]. Recently, the *PALB2* (partner and localizer of *BRCA2*) gene, which product, together with that of the *BRCA2* gene, is involved in DNA double strand break repair, has also attracted the attention of researchers. The loss of function of both alleles of this gene leads to Fanconi anemia, and the lack of function of one allele results in a higher incidence of cancers, especially breast cancer [19].

Germline mutations in *PALB2* have been identified in approximately 1–2% of familial breast cancer cases and 3–4% of HPC cases [20]. *PALB2* mutation analysis in 94 breast cancer patients without *BRCA1/2* mutations who had a family or personal history of PC showed a prevalence of 2.1% [20].

In summary, PC in HBOC is diagnosed later than breast or ovarian cancers, which gives opportunity to successful screening. Precise genetic testing may improve risk stratification and the detection of mutation may increase the number of effective, novel drugs that may be used in the therapy.

### Familial atypical multiple mole-melanoma (FAMMM)

Approximately 5–10% of cutaneous melanoma cases occur in families with an inherited predisposition [21]. In 1991, Lynch and Fusaro described the association between familial melanoma multiforme and PC [22]. It has been estimated that individuals from families affected by FAMMM syndrome have a 13–22-fold increased risk of developing PC compared to the general population [23].

A study [24] of the Melanoma Genetics Consortium (GenoMEL) showed that the presence of PC in individuals with familial melanoma is a strong predictor of a pathogenic *CDKN2A* variant. In the above-mentioned study, 190 out of 466 (41%) families had mutations in either *CDKN2A* or *CDK4*. Those included p16 protein mutations in 178 families, *CDK4* mutations in 5 families and *ARF* mutations 7 families. Among 66 families with familial melanoma in which PC occurred, as many as 49 (74%) had a *CDKN2A* mutation. No *ARF* or *CDK4* mutations were reported in this group.

The risk of PC is particularly increased in Dutch families with a *CDKN2A* mutation variant known as p16-Leiden, in which there is a deletion of 19 base pairs (c.225_243del19). In a study of 19 families with this specific mutation, PC was observed in 7. It has been estimated that 1 in 6 carriers (17%) of the p16-Leiden mutation will develop PC by 75 years of age [25].

It has been shown that there is a high risk of developing multiple PC tumors in carriers of the *CDKN2A*-p16-Leiden mutation. Therefore, after the detection of a primary tumor in patients with this mutation, it becomes very important to exclude the presence of a second synchronous tumor, and strict monitoring is necessary after partial pancreatectomy. Considering these findings, a total pancreatectomy offered to patients with early PC may be an appropriate treatment option in this group [26].

### Peutz-Jeghers syndrome (PJS)

Peutz-Jeghers syndrome is a rare hereditary condition characterized by mucocutaneous pigmentation and Peutz-Jeghers hamartomatous polyps, predominantly affecting the small intestine [27]. Most patients who meet the clinical diagnostic criteria for PJS have mutations in the *STK11*/*LKB1* gene. Its consequence is an individual predisposition to the development of many cancers, including breast, colorectal, pancreatic, gastric, small intestinal and cancers of the reproductive organs [28]. PC is the third most common cancer in patients with PJS, with a lifetime risk of 11–55% [27].

In a large homogeneous cohort involving 119 patients with PJS ascertained in sixteen different Italian centers, it was observed that the relative risk (RR) of PC development is the largest among all other neoplasms and reaches 139.7 (Cl 61.1–276.4). The cumulative risk of developing a PC is 2, 4.5, 18 and 55% at 40, 50, 60 and 65 years of age, respectively [29]. Interestingly, none of the PJS patients from Korea (*n* = 30) [30] or Japan (*n* = 14) [31] had PC.

### Hereditary non-polyposis colorectal cancer (Lynch syndrome)

Patients with Lynch syndrome (LS) are at increased risk of developing many cancers, predominantly colorectal and endometrial cancer. Other cancers, although less frequently, may also occur. It is an autosomal dominant inherited disease that is characterized by the presence of mutations in the genes involved in the processes of repair of mismatched DNA base pairs (MMR mismatch repair genes): *MLH1*, *MSH2*, *MSH6*, *PMS2* or deletions in *EpCAM* [32]. Initial data on the prevalence of *EpCAM* deletions as a cause of LS suggested a causative role in up to 2.8% of subjects [33]. Recent studies show that one of the *EpCAM* deletions (i.e. c.858 + 2478_*4507del) that was found in the Polish population [34] may occur in a comparable number to *MLH1* (e.g., c. 2041G > A) and *MSH2* (e.g. c.942 + 3A > T) mutations [35]. LS may be suspected by meeting the Amsterdam I or less strict Amsterdam II criteria and the Bethesda guidelines, but currently the diagnosis must be confirmed by a detection germline mutations or deletions [36].

The cumulative risk of PC in LS patients is approximately 3.7%, which translates into an 8.6-fold increase in the risk of development of PC compared to the general population [37]. Tumors seen in the course of disease often have characteristic medullary appearance with significant lymphocytic infiltration.

### Familial adenomatous polyposis (FAP)

FAP is an autosomal dominantly inherited syndrome with a germline mutation in the *APC* gene. The clinical manifestations of the mutation are multiple (> 100, usually hundreds or thousands) adenomatous polyps within the colon. Polyps may also appear in other parts of the gastrointestinal tract, e.g., in the stomach, duodenum, small intestine and biliary tract [38].

In earlier studies, the risk of PC in individuals with FAP was estimated to be four times higher than in the general population. The absolute lifetime risk of its occurrence was estimated at 2% [39]. Currently, due to the rare occurrence of tumors of the exocrine part of the pancreas in FAP patients, their genetic association is becoming dubious [38]. However, it should be kept in mind that almost all individuals with FAP develop duodenal polyps, of which 4–18% will progress to cancer by 75 years of age [40, 41]. Currently, prophylactic endoscopic polypectomy is a relatively safe and effective procedure to prevent the development of cancer in the duodenum, including the periampullary region directly associated with the head of the pancreas [42].

### Hereditary pancreatitis (HP)

Hereditary pancreatitis (HP) has been defined as pancreatitis occurring in 2 or more individuals in a family for 2 or more generations or pancreatitis associated with a mutation in the cationic trypsinogen *PRSS1* (protease serine 1) gene. It is an autosomal dominant disease. In most cases, it manifests with acute pancreatitis in childhood or chronic pancreatitis in early adolescence. In addition to *PRSS1* gene mutations, mutations of the serine protease inhibitor Kazal type 1 (*SPINK1*) and cystic fibrosis transmembrane conductance regulator (*CFTR*) genes are factors that cause or modify the course of the disease [43]. In a study carried out in 87 patients with HP, a specific gene mutation was found in 54 (62%) cases. The majority had defective *PRSS1* gene (34 patients). Nevertheless, *SPINK1* and *CFTR* mutations were also common (detected in 14 and 15 patients, respectively) [44].

Data on *SPINK1* mutations that lead to pancreatitis have shown inconsistent results on risk of PC. In a large Chinese cohort study, there was no relation between mutation in *SPINK1* and PC occurrence [Cox HR = 0.39 (0.14–1.04); *p* = 0.059] [45]. On the other hand, in a European study a mutation in *SPINK1* gene was associated with a 12-fold increase in the risk of PC [Cox HR 12.0 (3.0–47.8), *p* < 0.001] [46]. The reason for its discrepancy is not fully understood. One of the explanations might be different origins of study populations (i.e. Asians and Caucasians) that were evaluated in the studies. Furthermore, the mutations that were detected in those trials were different, which may suggest that certain variants are more likely to cause PC (e.g., c.101A > G), whereas others (e.g., c.194 + 2 T > C) lead rather to pancreatitis. Recent analyses from UK Biobank have not indicated increased PC risk in patients with the most common *CFTR* (c.1521_1523delCTT) mutation [OR = 1.2 (0.85–1.64) *p* = 0.26] [47]. The area requires further research, because even large scale meta-analyses provide conflicting results in that area showing modest increase (OR, 1.41; 95% CI, 1.07–1.84; *P* = 0.013) in the risk of PC in *CFTR* mutation carries, whereas no association between PC and *SPINK1* mutations (OR, 1.52; 95% CI, 0.67–3.45; *P* = 0.315) [48].

In a study conducted by EUROPAC (European Registry of Hereditary Pancreatitis and Pancreatic Cancer) in 2004, which included 112 families with HP from 14 countries, mutations in *PRSS1* gene were found in 81% of families included in the analysis. The cumulative risk of developing PC by 70 years of age after the onset of the disease is 44%, and the standardized incidence rate is 67% [49].

Recent studies of HP patients in the United States of America (US) [50] and Japan [51] have confirmed the high risk of PC in this group, but it was much lower than that in previous reports. The cumulative risk of cancer by the age of 70 in HP patients was only 7.2% in the US study and 22.8% in the Japanese study. According to the authors, explanations for this discrepancy in the results between the studies may include the different number of patients in the two studies who lived to 70 years of age, referral bias, a larger number of mutations related to HP that were previously unknown, and an increased number of tobacco smokers and a lifestyle modification associated with it. Tobacco smoking accelerates the development of PC by two decades, while alcohol remains only a risk factor for the development of pancreatitis [52]. As a preventive measure of PC in patients with HP-induced chronic pancreatitis presenting with chronic pain, some have advocated for early total pancreatectomy and islet autotransplantation [53].

### Li-Fraumeni syndrome (LFS)

LFS is a result of a germline autosomal dominant *TP53* gene mutation. Lack of *TP53* suppressor gene leads to the development of various neoplasm, typically: sarcomas, breast cancer, leukemia, adrenocortical cancer and brain tumors [54]. However, since the original observations it has been noted that several other cancers tend to occur more often in patients suffering from LFS. The risk of PC was also found to be elevated in LFS [55] and it was calculated that compared to subjects without *TP53* mutations, patients with positive *TP53* mutation have 7.3-fold increased risk in PC development [54].

The course of screening routine for PC in patients with LFS has not been established. It is considered that the introduction of screening in patients above 50 years of age may be a viable option, especially in the setting of history of PC in 1st or 2nd degree relative [56].

### Ataxia telangiectasia (AT)

Ataxia-telangiectasia is an autosomal recessive disease, which is a result of *ATM* gene mutations [57]. ATM is a serine/threonine kinase that phosphorylates p53 (a product of *TP53* gene). Lack of its function impairs the activity of p53. It is a rare condition, with worldwide occurrence between 1:40,000 and 1:300,000. The clinical picture consists of increased rates of cancers, immunological deficits and cerebellar dysfunction. According to recent clinical data, PC risk is more than 4 times higher even in heterozygous carriers of *ATM* gene mutation than in control subjects [OR 4.21 (3.24–5.47); *p* < 0.0001] [58]. With the advent of new diagnostic methods (e.g., whole genome and exome sequencing) 4–9% of ductal PCs were found to have mutations in *ATM* gene [59]. Despite that increased risk, there are no specific recommendations on screening for PC in patients with AT or *ATM* mutation carriers.

### Werner’s syndrome (WS)

WS is a rare (1:100,000) autosomal recessive disease resulting from loss-of-function of *WRN* gene [60]. Physiologically, *WRN* expression leads to increased activity of p53 and cell cycle arrest via p21CDKN1A and its absence may be responsible for accelerated carcinogenesis. The data on the incidence of PC in WS are scarce. There are several case reports describing aggressive clinical courses of the disease [61]. Additionally, it was noted that patients with WS develop PC earlier (52.3 ± 9 years) than patients with sporadic PC.

## Familial pancreatic cancer (FPC)

FPC is diagnosed when a family has 2 or more cases of PC in first-degree relatives, while no other hereditary cancer syndrome is observed [62]. The first reports on the increased risk of PC in relatives of people who already had this cancer were published in the 1970s. These observations were followed by several case-control and cohort studies, in which the risk of PC was estimated to be 2 to 5 times higher than in the general population, depending on the number of family members with this cancer [4]. These publications led to the foundation of a registry of families affected by PC in the USA [National Familial Pancreas Tumor Registry (NFPTR), Johns Hopkins] [63], Europe [European Registry of Familial Pancreatic Cancer and Hereditary Pancreatitis (EUROPAC)] [64] and several other developed countries, including Germany [65] or Italy [66]. Observations made by researchers at the NFPTR [63] showed a 9-fold increased risk of PC development in individuals who met the FPC criteria compared to a 1.8-fold increased risk in individuals from families with sporadic PC. The risk of developing PC in FPC increases depending on the number of affected family members. It is estimated that the risk varies from 4.6-fold for 2 PC cases in a family to 32-fold for 3 PC cases in a family. The lifetime risk of developing PC in FPC individuals is estimated at 18–38%. However, in a Danish national cohort of 27 families with FPC the risk of PC was estimated at 51%, which clearly emphasizes the contribution of genetic and environmental factors to the development of this cancer [67]. Similarly to cases of sporadic PC, in FPC individuals, exposure to tobacco smoke (both active and passive) was found to be the main modifiable risk factor for this cancer, leading to its earlier development [68]. Newly diagnosed diabetes mellitus is also a significant risk factor for cancer development in FPC families. In a study on a Japanese population [69], it was shown that the presence of 2 risk factors, smoking and diabetes mellitus, in an FPC individual increases the risk of PC by up to 10 times.

In FPC, cancer tends to occur more frequently in younger age groups than in the sporadic form. Importantly, the risk of developing PC also increases with the decreasing age of onset of the cancer in subsequent generations [70]. After a review of the genealogy of 106 families meeting the FPC criteria, it was found that from one generation to the next, the age of death from PC of successive descendants of these families is lower and the prognosis is worse because of the more aggressive form of the cancer. Offspring of parents in the FPC group died 10 years earlier as a result of PC. With this observation, it is now possible to predict the age of cancer onset in subsequent generations of members of such families [71].

Based on a detailed analysis of FPC group data, the first model for predicting its risk in subsequent family members (PancPRO) was developed [72]. As many as 80–90% of the genetic events leading to FPC remain unknown, and only 10–20% have a uniquely identifiable germline mutation. This makes it difficult to properly distinguish FPC from apparently sporadic ductal adenocarcinoma. In particular, inheritance can often be attributed to germline mutations of DNA repair and damage response genes and mutations in other classical cancer susceptibility genes, e.g., *CDKN2A* or *TP53* [73].

A large cohort (*n* = 515) developed by the Pancreatic Cancer Genetic Epidemiology (PACGENE) multicenter consortium has provided a comprehensive analysis of germline mutations occurring in four genes, *BRCA1* (1.2%), *BRCA2* (3.7%), *PALB2* (0.6%), and *CDKN2A* (2.5%), among familial pancreatic cancer probands [74]. Most recent results from German National Case Collection for Familial Pancreatic Cancer (FaPaCa) show that an identifiable potentially culpable germline mutation was found in 16.6% of patients with FPC. The incidence was the highest for *BRCA2* mutations, which was diagnosed in 6% of families [75]. A study conducted in Poland [76] has also revealed that founder mutations in the *BRCA1*, *PALB2*, and *CHEK2* genes are present in a small proportion of PC patients from families diagnosed with FPC. Furthermore, in the same research site it was shown that one of the founder mutation (c.657_661del) in *NBS1* was associated with nearly fourfold odds ratio [OR = 3.8 (1.68–8.6) 95% CI] for the incidence of PC in a large cohort of patients with PC [77]. As a result, it was determined that only small proportion of FPC is a resultant of high penetrance mutations in genes connected also with typical hereditary PC (e.g. *BRCA1, BRCA2, PALB2* and *CDKN2A*). That gave rise to further studies (e.g. the PANcreatic Disease ReseArch – PANDoRA) on the connection between FPC and more common gene variants, but with low penetrance, i.e. genome-wide association studies [78].

## Genome-wide association studies (GWAS)

In order to identify genetic background for PC, another approach has been introduced in the twenty-first century [79]. Due to increased yield in genetic testing, a concept of analyzing multiple (up to several millions) single nucleotide polymorphisms (SNPs) scattered in the whole genome or only in exosomes in a case-control study was introduced. Those studies provided some interesting results. Around 50 specific loci were identified as connected with the increased rate of PC, but the strength of this relation in a single SNP is low (OR rarely is higher than 1.5) [79]. Therefore, single SNP variation is not powered enough to become a marker of PC. Examples of loci associated with the highest association with the risk of PC in Caucasian and Asian populations have been shown in Table 2 [80–84]. The majority of data comes from retrospective analyses and they lack prospective validation. Further studies are warranted to provide as much clinical and genomic data as possible. Nevertheless, SNP seems to be very promising element of combined or multigenic risk scores [85]. Compared to clinical markers only, the inclusion of genetic-based factors led to significant increase in area under ROC in the 10-year risk estimation model (0.61 vs. 0.67, *p* = 2.91 × 10^− 7^) [86].

**Table 2:** Loci associated with the risk of development of PC [80–84]

Chromosome	Gene	SNP	Odds ratio (95% CI)	*P* value
**Caucasians**				
16q23.1	*BCAR1/CTRB1/CTRB2*	rs7190458	**1.46 (1.3–1.65)**	1.1 × 10^− 10^
1p36.33	*NOC2L*	rs13303010	**1.26 (1.19–1.35)**	8.00 × 10^−14^
7q32.3	*LINC-PINT*	rs6971499	**0.79 (0.74–0.84)**	3.00 × 10^− 12^
1q32.1	*NR5A2*	rs3790844	**0.77 (0.71–0.84)**	2.45 × 10^− 10^
**Asians**				
2p24.1	*APOB*	rs183117027	**2.34 (1.72–3.16)**	4.21 × 10^−08^
8p21.3	*DOK2*	rs2242241	**1.85 (1.50–2.27)**	4.34 × 10^−09^
19p13.12	*PKN1*	rs34309238	**1.77 (1.48–2.12)**	5.25 × 10^−10^
16p12.3	*GP2*	rs78193826	**1.46 (1.29–1.66)**	4.29 × 10^−09^

## Preventive regimens in HPC and FPC

Due to the lack of impact on population mortality or cost-effectiveness, there are no recommendations to support PC screening in the general population. This screening is currently recommended if the lifetime risk of PC is > 5% [87]. Historically, the screening program has targeted individuals who are at more than 10 times the risk of developing PC compared with the general population. This risk level has generally been seen in family members with ⩾3 first-degree relatives with PC, individuals with FAMMM (p16 mutations), PJS, and hereditary pancreatitis [88].

In 2011, a 49-member multidisciplinary international expert consortium CAPS (International Cancer of the Pancreas Screening) met for the first time to discuss PC screening [89]. In 2018, CAPS [90] presented updated recommendations for PC screening. In the document experts underlined the importance of early detection of PCs. Therefore, it is crucial to single out individuals at the highest risk of carcinogenesis. Pancreatic surveillance should be performed in a research setting, including experienced multidisciplinary staff. Patients with *LKB1*/*STK11* or *CDKN2A* mutations should be screened irrespectively of family history toward PC. In case of other HPC (*BRCA2*, *BRCA1*, *PALB2*, *ATM*, *MLH1*, *MSH2*, or *MSH6*) and familial PC, screening should be introduced in patients with the history of at least one first-degree relative with diagnosed PC. The screening should start no earlier than 50 years of age in FPC, 40 years of age in case of PJS and FAMMM. Patients with other detected mutations should be screened around 45 years of age. Importantly, regardless of genetic background, the screening should be started 10 years earlier than the youngest affected blood relative. As an imaging modality authors suggested magnetic resonance imaging (MRI) or endoscopic ultrasound (EUS), which should be repeated annually. Summary of recommendations is presented in Table 3.

**Table 3:** Summary of the main recommendations of the 2019 International Cancer of the Pancreas Surveillance (CAPS) Consortium [90]

Target population		
Peutz-Jeghers syndrome (carriers of a germline *LKB1*/*STK11* gene mutation) Germline *CDKN2A* mutation Germline *BRCA2*, *BRCA1*, *PALB2*, *ATM*, *MLH1*, *MSH2*, or *MSH6* gene mutation with at least one affected first-degree blood relative Individuals who have at least one first-degree relative with PC who in turn also has a first-degree relative with PC (FPC kindred)		
The onset of screening		
FPC kindred (without a known germline mutation)	Start at age 50 or 55 or 10 years younger than youngest affected blood relative	
Mutation carriers: for *CDKN2A*, Peutz-Jeghers syndrome,	Start at age 40 or 10 years younger than the youngest affected blood relative	
Mutation carriers: for *BRCA2*, *ATM*, *PALB2*, *BRCA1*, *MLH1*/*MSH2*	Start at age 45 or 50 or 10 years younger than the youngest affected blood relative	
Method of screening		
At baseline	MRI/MRCP + EUS + fasting blood glucose and/or HbA1c	
Follow-up	Alternate MRI/MRCP and EUS (no consensus if and how to alternate)	
	Routinely test fasting blood glucose and/or HbA1c	
On indication	Serum CA 19–9	If suspected features on imaging
	EUS-FNA only	Solid lesions of ≥5 mm
		Cystic lesions with suspected features
		Asymptomatic MPD strictures (regardless of tumor presence)
	CT only	Solid lesions, regardless of size
		Asymptomatic MPD strictures of unknown etiology (without tumor)
Intervals		
12 months	If no abnormalities, or only non-concerning abnormalities (e.g., pancreatic cysts without suspected features)	
3 or 6 months	If suspected abnormalities for which immediate surgery is not indicated	

Abbreviations

*CA19–9* Carbohydrate antigen 19–9

*CT* Computer tomography

*EUS* Endoscopic ultrasound

*FNA* Fine-needle aspiration

*FPC* Familial pancreatic cancer

*MPD* Main pancreatic duct

*MRCP* Magnetic resonance cholangiopancreatography

*MRI* Magnetic resonance imaging

*PC* Pancreatic cancer

In 2019, the Italian Association for the Study of the Pancreas (AISP) published modified surveillance criteria for FPC and HPC individuals included in the Italian Registry of Families at Risk of Pancreatic Cancer (IRFARPC), established in 2015 [66]. Recently, updated American Gastroenterological Association (AGA) guidelines for PC screening indications in high-risk individuals have been published in 2020 [91]. A comparative summary of the major recommendations of the 3 organizations CAPS, AGA, and AISP is presented in Table 4. Compared to the CAPS, the difference in the AISP and AGA recommendations is the inclusion of individuals with hereditary pancreatitis to the screening group. Furthermore, AISP recommends screening 10 years earlier than CAPS. AGA recommendations are similar to CAPS, but patients with PJS should be screened earlier - around 35 years of age. The scope and frequency of imaging studies are similar in all mentioned-above recommendations. The issue that still requires addressing in the view of the recommendation is their cost-effectiveness. Definitely this area requires further assessment.

**Table 4:** Summary of the main recommendations on screening for PC (AISP, CAPS, AGA) [66, 90, 91]

	CAPS	AGA	AISP
Target population			
PJS (*LKB1*/*STK11*)	+	+	+
FAMMM (*CDKN2A*)	+	+	+
HBOC (*BRCA1*, *BRCA2*, *PALB2*)	+	+	+
LS (*MLH1*, *MSH2*, or *MSH6*)	+	+	+
AT (ATM*)*	+	+	–
FPC	+	+	+
HP (*PRSS1*, *SPINK1*, *PRSS2*)	?	+	+
The onset of screening (patients’ age)			
PJS	40	35	30
FAMMM	40	40	30
HBOC	45 or 55^a^	50^a^	40^b^
LS	45 or 55^a^	50^a^	40^b^
AT	45 or 55^a^	50^a^	–
FPC	50 or 55^a^	50^a^	45^a^
HP	–	40	40^b^
Method of screening			
MRI/MRCP	+	+	+
EUS	+	+	+
HbA1c	+		
Intervals			
If no abnormality	12 M	12 M	12 M
If abnormality	3 or 6 M	3 or 6 M	Mf

Abbreviations

*CAPS*- Cancer of the Pancreas Surveillance Consortium

*AGA* American Gastroenterological Association

*AISP* Italian Association for the Study of the Pancreas AISP

*PJS* Peutz -Jeghers syndrome

*FAMMM* Familial atypical multiple mole melanoma

*HBOC* Hereditary breast-ovarian cancer syndrome

*LS* Lynch syndrome

*AT* Ataxia-teleangiectasia syndrome

*HP* Hereditary pancreatitis

*FPC* Familial pancreatic cancer

*M* Months

*Mf* More frequently in high-risk patients

^a^ 10 years less than age of the youngest affected family member

^b^ 5 years less than age of the youngest affected family member

The reduction in the incidence of PC should focus on a healthy lifestyle [92] and modifiable risk factors. Cancer prevention [93] and early detection of lesions may be effective in the reduction of mortality caused by PC. In case of HPC and FPC, attention should be given to a thorough physical examination and anamnesis. As a result, high-risk patients may be singled out and undergo screening programs. As mentioned in the introduction, the heritability in PC might be detected in up to 20% of patients. It seems that further studies on the genetic-based mechanisms are necessary to find patients with increased risk of PC. Currently, great effort is put on finding novel loci responsible for an increased PC risk. Further studies should focus on incorporation of results of GWAS studies into multifactorial risk estimations and finally an attempt should be made to validate them in a clinical outcome trial.

## Conclusions

In recent years, a large body of evidence has emerged supporting the influence of genetic factors on the development of PC. Selection in the groups of individuals who have a familial predisposition to PC through the development of genetic counseling is crucial in the fight against this cancer. Recent guidelines of scientific societies dealing with PC have selected groups of people who should be periodically screened. Currently, it seems that only increased screening and primary prevention may offer a chance for early cancer detection and effective therapeutic intervention. Nevertheless, the scope, methods used in screening programs and their cost-effectiveness require further analyses.

## Data Availability

Not applicable.

## Abbreviations

AGA: American Gastroenterological Association
AISP: Italian Association for the Study of the Pancreas
AT: Ataxia-teleangiectasia syndrome
*ATM*: Ataxia telangiectasia mutated
*BRCA1*: Breast cancer 1
*BRCA2*: Breast cancer 2
CA19–9: Carbohydrate antigen 19–9
CAPS: Cancer of the Pancreas Surveillance Consortium
*CDKN2A*: Cyclin-dependent kinase inhibitor 2A
CT: Computer tomography
EUS: Endoscopic ultrasound
FAMMM: Familial atypical multiple mole melanoma
FNA: Fine-needle aspiration
FPC: Familial pancreatic cancer
GWAS: Genome-wide association studies
HbA1c: Hemoglobin A1c
HBOC: Hereditary breast-ovarian cancer syndrome
HP: Hereditary pancreatitis
HPC: Hereditary pancreatic cancer
LFS: Li-Fraumeni syndrome
LS: Lynch syndrome
M: Months
Mf: More frequently in high-risk patients
*MLH1*: mutL homolog 1
MPD: Main pancreatic duct
MRCP: Magnetic resonance cholangiopancreatography
MRI: Magnetic resonance imaging
*MSH2*: mutS homolog 2
*MSH6*: mutS homolog 6
*PALB2*: Partner and localizer of *BRCA2*
PC: Pancreatic cancer
PJS: Peutz Jeghers syndrome
*STK11*: Serine/threonine kinase 11
WS: Werner’s syndrome
